# A DMAHDM–herbal hybrid gargle for orthodontic-associated complications via oral microbiota regulation, inflammation inhibition, and enamel protection

**DOI:** 10.1016/j.mtbio.2026.103372

**Published:** 2026-06-22

**Authors:** Yuqing Zhang, Bo Wang, Ce Bian, Chaoran Yu, Mengyao Zhu, Yiman Guo, Honglei Yue, Wenting Yu, Yuxing Bai, Ning Zhang

**Affiliations:** a Department of Orthodontics, Beijing Stomatological Hospital and School of Stomatology, Capital Medical University, No.9 Fanjiacun Road, Fengtai District, Beijing, 100070, PR China; b Department of Stomatology, The First Affiliated Hospital of Dalian Medical University, No. 222 Zhongshan Road, Dalian, Liaoning, 116000, PR China; c Beijing Institute of Dental Research, Beijing Stomatological Hospital, Capital Medical University, No. 9 Fanjiacun Road, Fengtai District, Beijing, 100070, PR China

**Keywords:** Microbiome regulation, Enamel demineralization, Anti-inflammatory, Antibacterial, Orthodontic treatment

## Abstract

Orthodontic appliances are indispensable for achieving therapeutic efficacy, but their complex and irregular architecture compromises oral hygiene, increasing susceptibility to dental caries and periodontal diseases. Conventional gargles primarily target antibacterial effects; however, their prolonged use may disrupt the balance of the oral microbiota, and remains insufficient for controlling periodontal inflammation and preventing enamel demineralization. Herein, a bioactive hybrid gargle integrating *Dimethylaminohexadecyl methacrylate* (DMAHDM) and *Lonicera japonica* extract (LJE) was developed. The combined use significantly downregulated the expression of pro-inflammatory cytokines and attenuated alveolar bone loss via activation of the peroxisome proliferator-activated receptor (PPAR) signaling pathway. Moreover, in addition to its potent antibacterial activity, this novel gargle exhibits a distinctive capacity to regulate the oral microbiota by modulating the relative abundances of *Bacteroidetes* and *Actinobacteria*, thereby promoting a more balanced and stable microbial community. It also effectively inhibited enamel demineralization, preserved calcium and phosphorus content, and maintained enamel hardness, while prolonged exposure exerted no adverse influence on the mechanical properties of orthodontic materials. The biocompatibility over a 60-day period was also systematically evaluated and confirmed. Collectively, these findings highlight the novel hybrid gargle as a promising therapeutic paradigm for the clinical management of orthodontic-associated complications, including periodontal diseases, dental caries, and enamel demineralization.

## Highlights

• DMAHDM–Herbal hybrid gargle for orthodontic-associated complications.
• Suppresses multispecies biofilms and modulates oral microbiota.
• Activates PPAR signaling and attenuates alveolar bone resorption.
• Preserves enamel Ca/P content and mechanical integrity.
• Demonstrates long-term biocompatibility and material stability.

## 1 Introduction

Orthodontic treatment achieves therapeutic efficacy through the use of orthodontic appliances. Nevertheless, their complex and irregular architecture hinders effective oral hygiene, thereby increasing susceptibility to dental caries [1] and periodontal diseases [2]. Previous studies have reported that among adolescents undergoing orthodontic treatment, the prevalence of enamel white spot lesions—an early manifestation of dental caries resulting from microbial acid production and disrupted enamel mineralization dynamics [3,4]—can be as high as 34.6% [5]. Additionally, other investigations have demonstrated an elevated risk of periodontal disease during fixed orthodontic treatment [6,7], a condition characterized by progressive destruction of the soft–hard tissue interface of the periodontium [8]. Therefore, the development of effective plaque control strategies is essential for the prevention and management of orthodontic related complications.

The use of antimicrobial-containing gargles to control dental plaque is a common and effective adjunctive approach for plaque management [9]. Currently, most commercial gargles rely on broad-spectrum antimicrobial agents, such as chlorhexidine and cetylpyridinium chloride (CPC). Although these formulations can effectively suppress plaque accumulation, they are associated with several notable drawbacks. For instance, chlorhexidine has been reported to cause staining of clear aligners and even tooth surfaces [10], leading to significant aesthetic concerns. More importantly, conventional antimicrobial agents may severely disrupt the physiological balance of the oral microbiota, thereby inducing oral microbial dysbiosis [11]. Consequently, there is an urgent need to identify novel antimicrobial agents and to develop novel gargles with integrated and multifunctional therapeutic effects [12,13].

*Dimethylaminohexadecyl methacrylate* (DMAHDM), a recently developed quaternary ammonium salt (QAS) monomer, exhibits the strongest antibacterial activity among QAS compounds due to its sixteen-carbon alkyl chain structure [14]. This potent antibacterial effect is primarily attributed to its cationic groups that electrostatically interact with negatively charged bacterial membranes, allowing the long hydrophobic chain to penetrate the membrane and induce bacterial lysis and death. Previous studies have successfully incorporated DMAHDM into various dental materials, where it has consistently demonstrated robust and durable antibacterial efficacy [15,16]. Notably, additional evidence indicates that DMAHDM can reduce the proportion of acidogenic bacteria within biofilms [17,18], suggesting a potential oral microbiota–regulating capability that may support its long-term applicability. To further enhance antimicrobial performance, delay the development of bacterial resistance, and address periodontal health challenges, the incorporation of multiple bioactive agents represents a promising strategy [19]. Herbal therapeutics offer valuable opportunities for identifying such complementary agents. *Lonicera japonica* (honeysuckle), the flower bud or blossom of the honeysuckle vine, is an edible herbal medicine renowned for its potent antibacterial and anti-inflammatory properties. *Lonicera japonica* extract (LJE) has been shown to inhibit the growth of common oral pathogens, including *Streptococcus mutans* and *Porphyromonas gingivalis* [20,21], highlighting their effectiveness as natural antibacterial agents. Moreover, as a traditional anti-inflammatory remedy, LJE has been demonstrated to reduce intracellular reactive oxygen species (ROS) production and lipid peroxidation *in vitro*, while significantly downregulating inflammatory mediators such as IL-1β, IL-6, and TNF-α compared with controls—highlighting its therapeutic potential for the prevention and treatment of periodontitis [22].

**Fig. 1:**
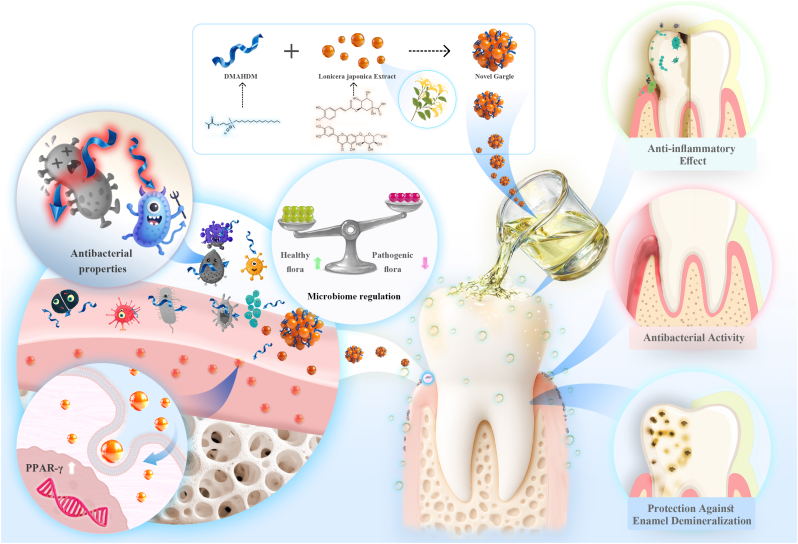
Conceptual illustration of the formulation, biological functions, and action mechanisms of the DMAHDM–herbal hybrid multifunctional gargle. The novel gargle was formulated by combining DMAHDM with *Lonicera japonica* extract (LJE). Upon application, the formulation exerts multiple oral health–promoting effects through distinct mechanisms: (1) antibacterial activity, in which DMAHDM disrupts the membranes of oral pathogenic bacteria to inhibit plaque biofilm formation; (2) microbiome regulation, promoting the growth of healthy flora while suppressing pathogenic species to restore oral microbial balance; (3) anti-inflammatory effects, in which LJE alleviates gingival inflammation and attenuates alveolar bone resorption through activation of the PPAR-γ signaling pathway; and (4) protection against enamel demineralization, thereby contributing to the prevention of dental caries.

Therefore, by integrating the complementary properties of DMAHDM and *Lonicera japonica* extract, this study developed a novel multifunctional gargle. This formulation exhibits several key features: (1) it effectively suppresses the formation and metabolic activity of multispecies plaque biofilms while exerting a critical oral microbiota regulating effect; (2) it alleviates periodontitis by activating the peroxisome proliferator-activated receptor (PPAR) signaling pathway, thereby significantly reducing alveolar bone resorption; and (3) it effectively preserves enamel calcium and phosphorus contents in in vivo models, maintaining the structural integrity and mechanical stability of enamel. In addition, its biocompatibility was systematically validated through a series of *in vitro* and *in vivo* evaluations (Fig. 1).

## 2 Results and discussion

### 2.1 Synthesis and characterization of DMAHDM and *Lonicera japonica* extract

**Fig. 2:**
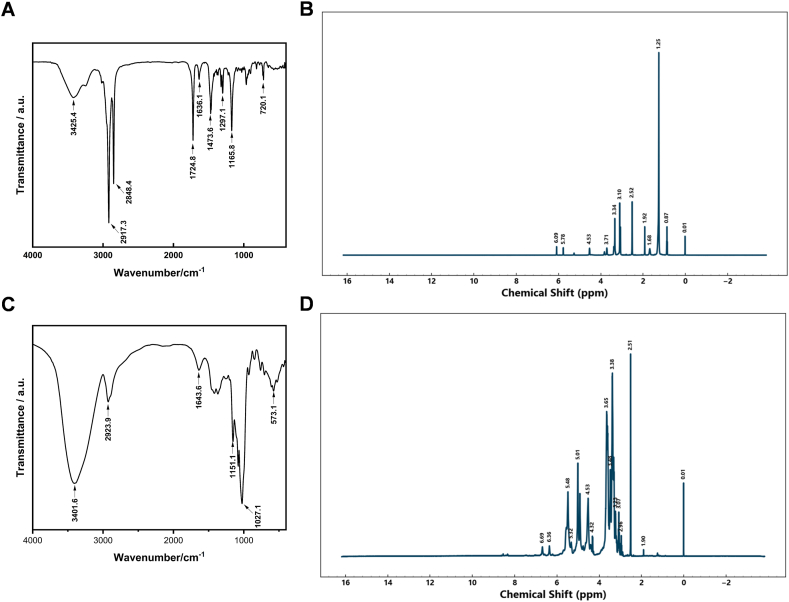
Characterization of the novel gargle components. **A.** Fourier-transform infrared (FTIR) spectrum of DMAHDM. **B.**^1^H nuclear magnetic resonance (^1^H NMR) spectrum of DMAHDM. **C.** FTIR spectrum of LJE. **D.**^1^H NMR spectrum of LJE.

The Fourier Transform Infrared Spectroscopy (FTIR) spectrum of DMAHDM (Fig. 2A) exhibited characteristic bands at 3452 cm^−1^ (O–H stretching from adsorbed moisture), 2917–2848 cm^−1^ (C–H stretching of long alkyl chains), 1724 cm^−1^ (ester C=O), and 1638 cm^−1^ (methacrylate C=C), consistent with the successful synthesis of DMAHDM. The ^1^H NMR spectrum (Fig. 2B) further supported this structure, with resonances at *δ* 6.09 and 5.57 ppm (vinylic = CH_2_), 4.08 ppm (–O–CH_2_–), 3.37 ppm (N^+^–CH_2_–), 1.84 ppm (C=C–CH_3_), 1.28 ppm (alkyl –CH_2_–), and 0.87 ppm (terminal –CH_3_); a narrow singlet attributable to N^+^(CH_3_)_2_ is typically observed near *δ* 3.1–3.3 ppm.

The FTIR spectrum of LJE (Fig. 2C) exhibited a broad O–H band at 3401 cm^−1^, aromatic C=C stretching at 1643 cm^−1^, and glycosidic C–O–C/C–O bands at 1151–1027 cm^−1^, while a weak feature near 573 cm^−1^ was assigned to low-frequency skeletal vibrations. The ^1^H NMR spectrum (Fig. 2D) showed aromatic resonances at *δ* 6.8–6.1 ppm, an anomeric signal at *δ* ∼5.1 ppm, and sugar-related multiplets at *δ* 4.3–3.2 ppm, consistent with the structural features of polyphenolic glycosides.

Together, the FTIR and ^1^H NMR data confirm the successful synthesis of DMAHDM and are consistent with the presence of chlorogenic acid and luteoloside as principal bioactive constituents in LJE.

### 2.2 Antibacterial activity and microbiome regulation of the novel gargle

Inhibition of dental plaque biofilm formation is one of the most critical functions of a gargle. In this study, we investigated the feasibility of incorporating DMAHDM as the primary antibacterial agent in the formulation. Based on the results of the biocompatibility assessment, DMAHDM was tested at mass fractions of 0.1 wt% and 0.2 wt% in antibacterial assays. To closely mimic the *in vivo* oral environment, a human whole-saliva-derived polymicrobial biofilm model was employed to evaluate the antibacterial efficacy of the novel gargle.

**Fig. 3:**
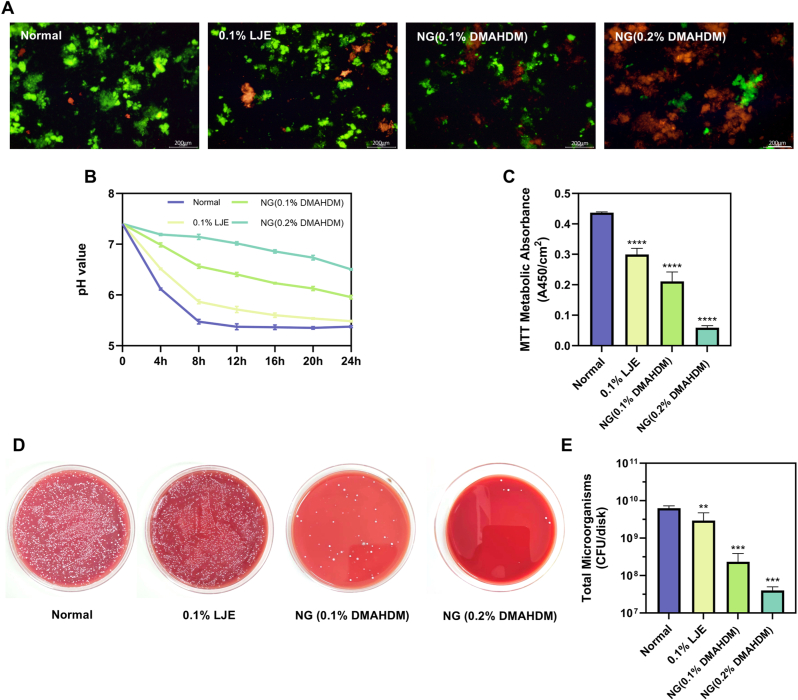
Evaluation of antibacterial activity of the novel gargle. **A.** Live/dead fluorescence imaging. **B.** pH changes of the bacterial suspension over 24 h. **C.** MTT assay results of biofilm metabolic activity. **D.** Representative total microorganism CFU plate images. **E.** Quantitative analysis of total microorganism CFU counts. Data in Fig. 3C and E are presented as mean ± standard deviation (s.d.). For all experiments, n ≥ 3. Statistical significance was analyzed using one-way analysis of variance (ANOVA) followed by Tukey's post hoc test. *p* < 0.05 (*), *p* < 0.01 (**), *p* < 0.001 (***), *p* < 0.0001 (****).

As shown in Fig. 3A, the incorporation of DMAHDM markedly reduced the green fluorescence signal corresponding to viable bacteria, while simultaneously increasing the red fluorescence signal representing dead bacteria, with the most pronounced bactericidal effect observed at a mass fraction of 0.2 wt%. As illustrated in Fig. 3B, the addition of DMAHDM also significantly slowed the rate of pH decline in the bacterial suspension and reduced the metabolic activity of the biofilm (Fig. 3C). Among all groups, the 0.2 wt% DMAHDM formulation exhibited the lowest metabolic activity, showing a significant decrease compared with the control. Fig. 3D presents the representative total microorganism colony-forming units (CFU) plate images, whereas Fig. 3E provides the corresponding quantitative analysis. Collectively, these results indicate that the novel gargle containing 0.2 wt% DMAHDM exhibits optimal antibacterial efficacy against polymicrobial plaque biofilms.

DMAHDM has previously been incorporated into various orthodontic materials, including dental adhesives [23], elastic ligatures [18], and retainers [24], to impart antibacterial properties. In the present study, DMAHDM was applied to a gargle formulation for the first time, and the experiments confirmed its strong antibacterial efficacy as the principal active component.

**Fig. 4:**
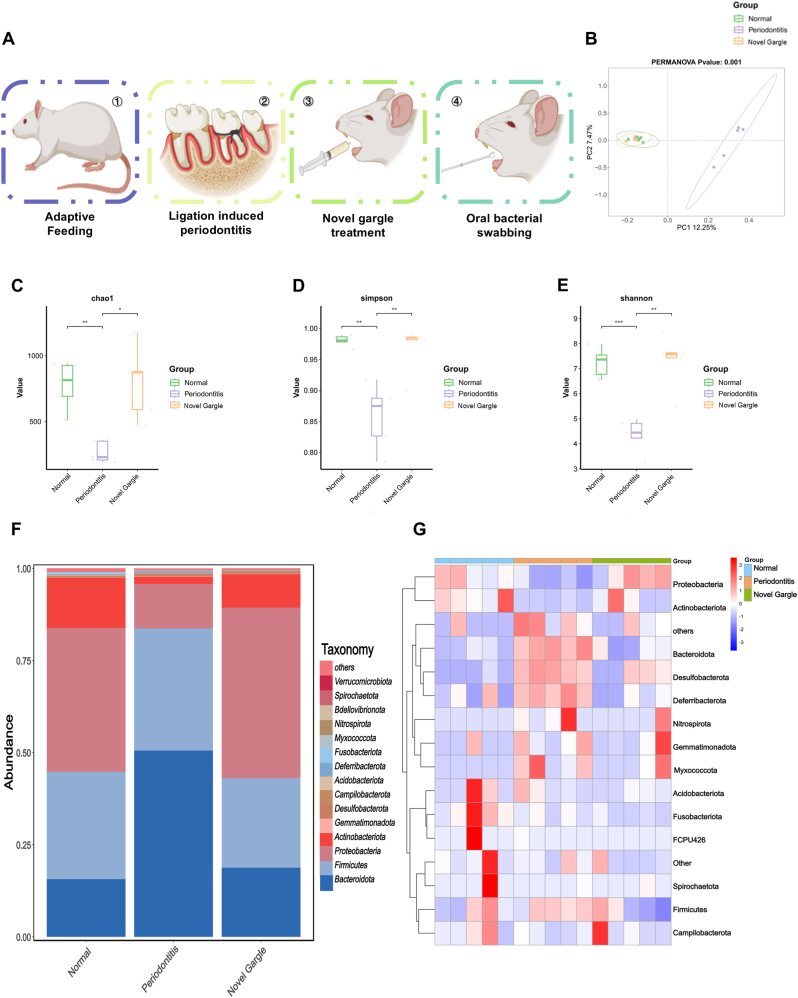
Evaluation of microbiome regulation of the novel gargle. **A.** Schematic illustration of the *in vivo* experimental workflow for evaluating oral microbiota regulation. **B.** Principal coordinate analysis (PCoA) based on Bray–Curtis distances. **C–E.** α-Diversity indices (Chao1, Simpson, and Shannon). **F.** Relative abundance of the dominant bacterial phyla. **G.** Taxonomic composition heatmap showing that the oral microbiome of the novel gargle group resembled the normal group. Data in Fig. 4C–E are presented as box plots showing the median, interquartile range, and individual data points. For all *in vivo* experiments, n = 5. Statistical significance was analyzed using one-way analysis of variance (ANOVA) followed by Tukey's post hoc test. *p* < 0.05 (*), *p* < 0.01 (**), *p* < 0.001 (***), *p* < 0.0001 (****).

Given that dental plaque biofilms are characterized by structural resilience and microbial complexity [25], and that the oral cavity inherently harbors a commensal microbiota, complete bacterial eradication is not the most desirable therapeutic goal. Instead, regulating the microbial community to maintain ecological balance is of greater clinical importance than pursuing excessive antibacterial activity. Therefore, 16S ribosomal RNA (16S rRNA) high-throughput sequencing was conducted to investigate the microbiome-regulating effects of the novel gargle (Fig. 4A).

The Venn diagram (Supplementary Fig. 1A) and the corresponding petal plot (Supplementary Fig. 1B) of shared species showed that the periodontitis group harbored fewer unique microbial species than the control and gargle groups. Principal coordinates analysis (PCoA) revealed that the microbial composition of the gargle group was more similar to that of the control group than to that of the periodontitis group (Fig. 4B). Alpha-diversity analysis based on the Chao1, Simpson, and Shannon indices (Fig. 4C–E), together with beta-diversity analysis (Supplementary Fig. 1C), demonstrated that microbial richness and diversity were significantly lower in the periodontitis group than in the control and gargle groups. These findings are consistent with previous reports [26] showing that oral microbiome diversity decreases during periodontitis. Treatment with the novel gargle partially restored microbial diversity and tended to reestablish microbial equilibrium.

At the phylum level (Fig. 4F and Supplementary Fig. 1D), the four dominant bacterial phyla across all samples were *Bacteroidetes*, *Firmicutes*, *Proteobacteria*, and *Actinobacteria*. During periodontitis, the relative abundance of *Bacteroidetes* increased, whereas *Actinobacteria* decreased, suggesting that periodontitis promoted a shift toward a dysbiotic microbial community. *Bacteroidetes* includes several disease-associated taxa [27], such as *Porphyromonas gingivalis*, a key pathogen of periodontitis [28]. In contrast, the gargle-treated group maintained stable *Actinobacteria* abundance and showed a reduction in *Bacteroidetes* levels. Although *Bacteroidetes* did not fully return to control levels, the overall microbial composition in the gargle group closely resembled the healthy baseline (Fig. 4G).

These results together suggest that the novel gargle may contribute to the rebalancing of the oral microbiome disrupted during periodontitis. This regulatory capability, which differs fundamentally from the non-selective antibacterial effects of conventional agents such as chlorhexidine or CPC, is particularly valuable for maintaining oral ecological balance and may help reduce the risk of enamel demineralization [29], periodontitis, and related systemic diseases [30]. Although the hybrid formulation was designed based on the complementary biological characteristics of DMAHDM and LJE, the interaction effects between varying ratios of the two components were not systematically investigated, and whether they exert synergistic or additive effects on specific biological endpoints remains to be fully elucidated, representing a limitation of the current study.

### 2.3 Anti-inflammatory effects and mechanism of the novel gargle

Periodontitis is one of the most common complications associated with orthodontic treatment [31]. The most effective means of preventing it remains proper tooth brushing. However, a considerable proportion of orthodontic patients are adolescents whose self-discipline and compliance are often limited. Therefore, we aimed to simplify prevention by endowing the novel gargle with intrinsic anti-inflammatory properties. *Lonicera japonica* is a traditional Chinese medicinal herb whose active constituents have been widely reported to exhibit potent anti-inflammatory effects [32,33].

To verify its suitability for incorporation into the gargle, the anti-inflammatory potential of LJE was first evaluated *in vitro*. After 24 h of LPS stimulation, the expression of *Tnfa* and *Il6* in RAW264.7 cells was markedly increased compared with the normal control (Supplementary Fig. 2A–B). When 0.03 wt% LJE was added, the expression levels of both inflammatory genes were significantly reduced, demonstrating a pronounced anti-inflammatory effect. Subsequent enzyme-linked immunosorbent assay (ELISA) results (Supplementary Fig. 2C–F) corroborated the gene-expression findings. For convenience in formulation, 0.1 wt% LJE was selected for the *in vivo* gargle preparation.

**Fig. 5:**
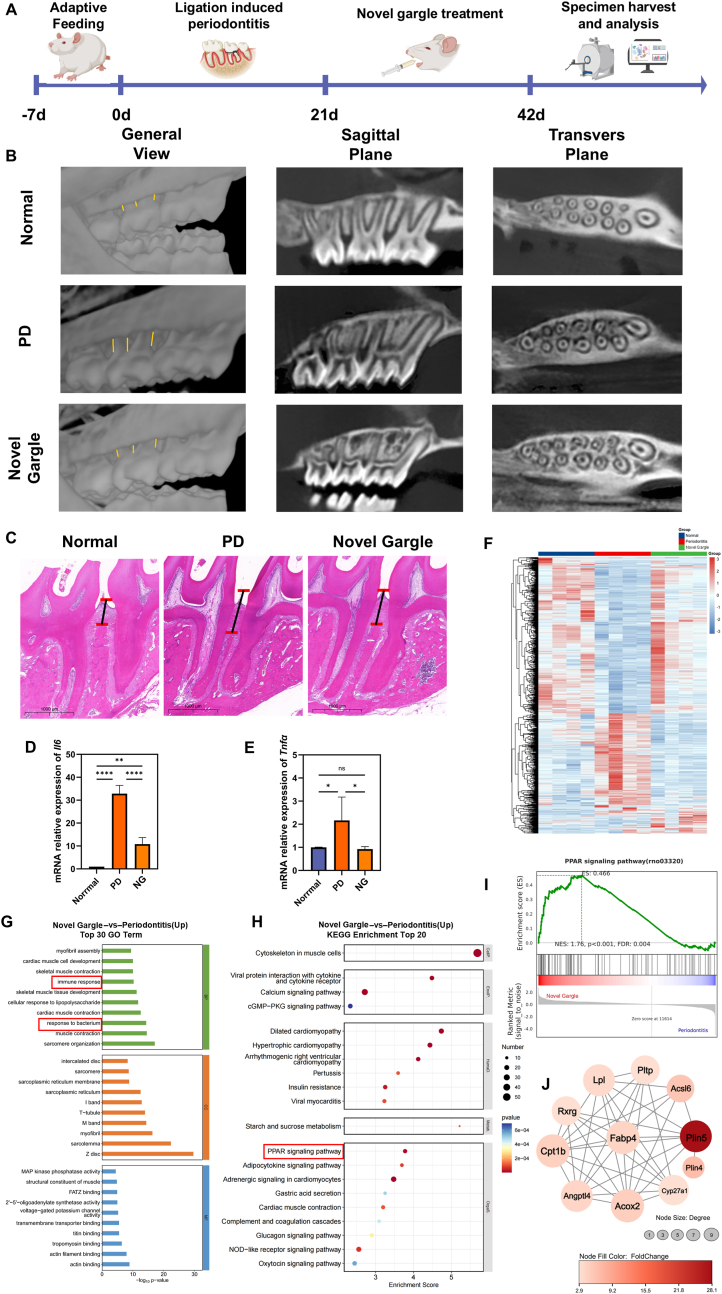
Evaluation of the anti-inflammatory effects and mechanism of the novel gargle. **A.** Schematic illustration of the *in vivo* experimental design for assessing the effects of the gargle and subsequent mechanistic investigations in a rat periodontitis model. **B.** Micro-computed tomography (micro-CT) of periodontal tissues in a ligature-induced periodontitis rat model. **C.** Hematoxylin–eosin (HE) staining images of periodontal bone histological sections. **D-E.** Quantitative PCR analysis of gingival tissues. **F.** Heat-map visualization displaying global transcriptional reprogramming following gargle treatment. **G.** Gene Ontology (GO) enrichment analysis. **H.** Kyoto Encyclopedia of Genes and Genomes (KEGG) pathway analysis. **I.** Gene-set enrichment analysis (GSEA). **J.** Protein–protein interaction (PPI) network. For *in vitro* experiments, n ≥ 3; for *in vivo* experiments, n = 4. Data are presented as mean ± standard deviation (s.d.). Statistical significance was analyzed using one-way analysis of variance (ANOVA) followed by Tukey's post-hoc test. *p* < 0.05 (*)*, p < 0.01* (**)*, p < 0.001* (***), *p* < 0.0001 (****).

A ligature-induced periodontitis model was established in rats, followed by simulated mouth-rinsing according to the experimental grouping. After 21 days of continuous treatment, all rats were sacrificed for analysis. Micro-computed tomography (micro-CT), gingival tissue collection, quantitative PCR, and RNA sequencing (RNA-seq) were conducted to examine gene-expression changes in gingival tissue under different treatments (Fig. 5A).

Micro-CT imaging (Fig. 5B) revealed obvious alveolar bone resorption at the mesial, middle, and distal sites of the maxillary second molar in the periodontitis group compared with the control group (Supplementary Fig. 3A). Consistently, quantitative Micro-CT analysis showed a significant reduction in bone volume fraction (BV/TV) and trabecular thickness (Tb.Th), accompanied by an increase in trabecular separation (Tb.Sp) in the periodontitis group (Supplementary Fig. 3B–D). Application of the novel gargle markedly attenuated alveolar bone loss, as evidenced by the partial restoration of these microstructural parameters, in agreement with histological observations of periodontal tissues (Fig. 5C). PCR analysis (Fig. 5D and E) further demonstrated that *Tnfa* and *Il6* expression levels in gingival tissues were markedly decreased in novel gargle-treated rats. These results confirm that LJE effectively alleviates periodontal inflammation. To further explore the underlying mechanism, RNA-seq analysis was performed.

Venn diagram analysis (Supplementary Fig. 3E) revealed that 3 Differential Expressed Genes (DEGs) were shared across all comparisons, with 268 DEGs uniquely identified in the Novel Gargle vs Periodontitis group, highlighting the specific gene expression changes induced by novel gargle treatment. Principal component analysis (PCA) further revealed a distinct clustering pattern for the periodontitis group, whereas the gargle-treated samples clustered closely with the normal controls (Supplementary Fig. 3F). Volcano-plot analysis showed that between the periodontitis and novel gargle groups, 377 DEGs were up-regulated and 313 were down-regulated (Supplementary Fig. 3G). The PCA clustering pattern was consistent with the DEGs and heatmap results (Fig. 5F), indicating a distinct transcriptional reprogramming following novel gargle treatment.

Gene Ontology (GO) enrichment analysis indicated that the significantly altered pathways were primarily associated with bacterial infection and immune-response processes (Fig. 5G). Kyoto Encyclopedia of Genes and Genomes (KEGG) pathway analysis suggested that up-regulated genes were enriched in the peroxisome proliferator-activated receptor (PPAR) signaling pathway (Fig. 5H and Supplementary Fig. 3H). To further elucidate the potential molecular mechanisms, gene set enrichment analysis (GSEA) was performed to assess pathway activation in the periodontitis and gargle groups (Fig. 5I). As expected, rats treated with the novel gargle exhibited marked enrichment of the PPAR signaling pathway. Protein–protein interaction (PPI) network analysis further supported these findings by revealing increased expression of anti-inflammatory genes associated with the PPAR pathway (Fig. 5J). However, further molecular validation is required to confirm pathway activation at the protein level.

Notably, previous studies have reported that luteoloside, one of the principal active components of *Lonicera japonica*, alleviates ulcerative colitis through activation of the PPAR signaling pathway [34], and other work demonstrated that *Lonicera japonica* mitigates hippocampal inflammation via the same mechanism [35]. Given these findings, it is plausible that the anti-periodontitis effect of *Lonicera japonica* extract in the novel gargle is mediated, at least in part, through modulation of the PPAR signaling pathway, although its downstream regulatory mechanisms warrant further investigation. It should be noted that the transcriptomic evidence presented here reflects pathway activation at the mRNA level only, and the absence of protein-level validation through Western blot analysis of PPARγ and its downstream targets, as well as functional confirmation via pharmacological inhibition, represents a limitation of the present study. Furthermore, the ligature-induced periodontitis model, while widely recognized as a gold-standard approach for recapitulating plaque-mediated periodontal inflammation and alveolar bone resorption, was not established in the direct presence of orthodontic appliances, limiting the direct extrapolation of these findings to the clinical orthodontic setting.

### 2.4 In vivo evaluation of enamel demineralization prevention

Dental enamel, the outermost layer of the tooth, is constantly subjected to various mechanical and chemical stresses [4]. Its remarkable mechanical resilience is crucial for maintaining normal oral function and overall dental health. Beyond its antibacterial effects, an ideal gargle should also provide protective benefits to the enamel surface. In this study, we further verified this protective capability.

**Fig. 6:**
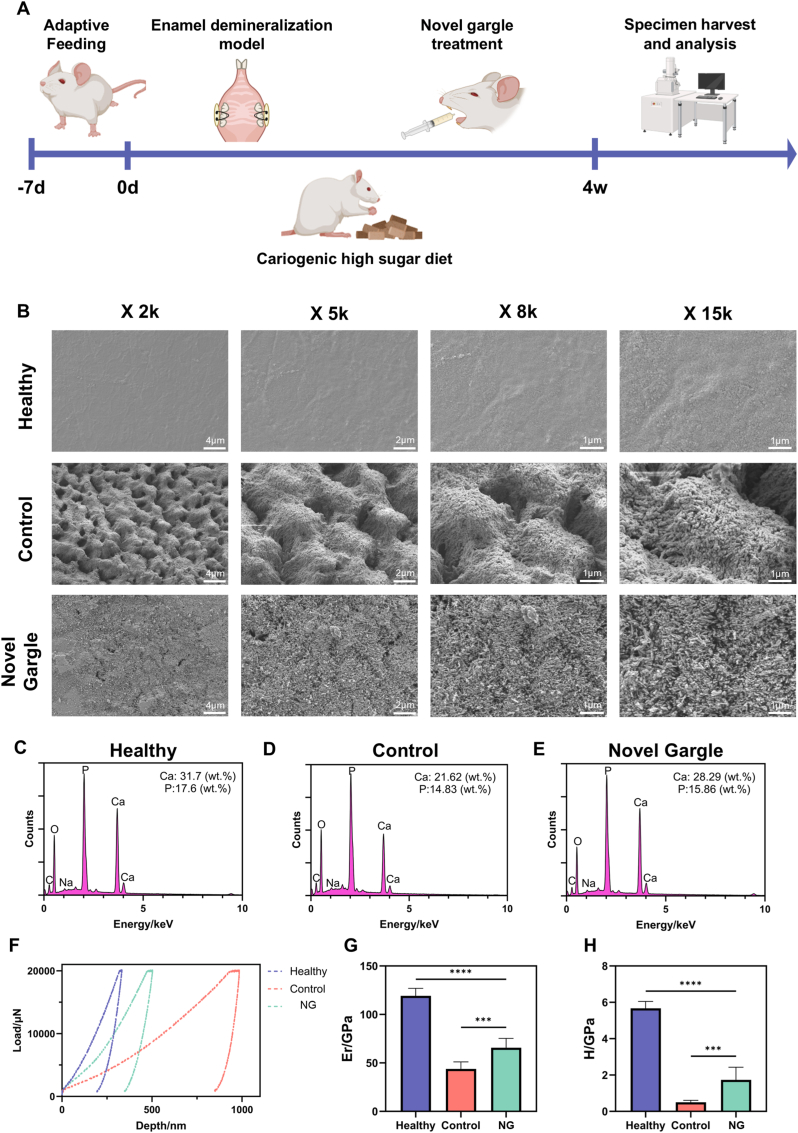
Evaluation of enamel-protective efficacy of the novel gargle. **A.** Schematic illustration of the *in vivo* experimental workflow for the enamel demineralization model and gargle treatment. **B.** Scanning electron microscopy (SEM) images of enamel surfaces. **C–E.** Energy-dispersive X-ray spectroscopy (EDS) analysis of enamel specimens, including elemental mapping, quantitative assessment of calcium (Ca) and phosphorus (P) contents, and representative EDS spectra. **F.** Nanoindentation load–displacement curves under a maximum load of 25 mN. **G.** Quantitative results for the elastic modulus (E). **H.** Surface hardness (H) measurements. n = 5. Data are presented as mean ± standard deviation (s.d.). Statistical significance was analyzed using one-way analysis of variance (ANOVA) followed by Tukey's post-hoc test. *p* < 0.05 (*)*, p < 0.01* (**)*, p < 0.001* (***), *p* < 0.0001 (****).

Although the morphology of rat molar enamel shares structural similarities with that of human enamel, a human enamel demineralization model was established in the oral cavities of rats to better approximate clinical conditions. The preventive efficacy of the novel gargle against enamel demineralization was evaluated from three perspectives: surface morphology, elemental composition, and mechanical properties (Fig. 6A).

As shown in Fig. 6B, a smooth and regular surface with well-aligned enamel prisms is characteristic of healthy enamel [36]. After four weeks of demineralization, partial dissolution of hydroxyapatite crystals occurred, and the enamel surface of the control group displayed numerous irregular pores, resulting in a rough and uneven texture. At higher magnification, enamel fibers appeared fractured and disorganized. In contrast, the enamel surface in the novel gargle group, although slightly rougher than that of healthy enamel, showed no obvious extensive fiber fracture or pronounced porosity.

Following enamel demineralization, elemental composition changed markedly, primarily reflected by decreases in calcium (Ca) and phosphorus (P) content [37]. Fig. 6C–E presents the elemental compositions of healthy enamel, the demineralized control group, and the novel gargle group. Both the control and 0.2 wt% DMAHDM groups exhibited reduced Ca and P contents compared with healthy enamel. However, the reductions in the control group (Ca: 21.62 wt%; P: 14.83 wt%) were more pronounced than those in the gargle group (Ca: 28.29 wt%; P: 15.86 wt%).

Loss of Ca and P often leads to deterioration in mechanical integrity. To evaluate this, nanoindentation tests were conducted to determine enamel surface mechanical parameters in each group. As shown in Fig. 6F, after four weeks of demineralization, indentation testing under a 25 mN load revealed that both the control and novel gargle groups had deeper indentations than healthy enamel, while the control group displayed the greatest depth. Fig. 6G and H further demonstrate that the elastic modulus (Er) and hardness (H) of enamel in the control group were significantly lower than those in the novel gargle group.

Overall, treatment with the novel gargle led to notable improvements in both elastic modulus and hardness compared with the demineralized control group, accompanied by higher Ca and P retention and less decline in mechanical performance. These findings suggest that the novel gargle effectively slows enamel demineralization, mitigates mechanical deterioration, and provides substantial protection against mineral loss. Nevertheless, the precise mechanism underlying this protective effect was not directly investigated in the present study, and the respective contributions of DMAHDM and LJE to enamel protection remain to be fully elucidated.

### 2.5 Effect of the novel gargle on the mechanical properties of clear aligners

Currently, orthodontic treatment is primarily conducted using either fixed appliances or clear aligners. As a modern orthodontic technology, the clinical effectiveness of clear aligners is largely determined by the mechanical properties of their thermoplastic materials [38]. However, existing cleaning solutions and disinfectants have been reported to adversely affect the mechanical stability of aligners [39]. Therefore, it was essential to verify that the novel gargle would not pose similar risks. To this end, a series of experiments were designed to evaluate the effects of prolonged immersion on the mechanical performance of clear aligners (Supplementary Fig. 4A–B). TPU and PETG were selected as representative aligner materials in this study, as they are currently the two most widely used polymers in the fabrication of clear aligners in clinical practice.

As shown in Supplementary Fig. 4C–D, the impact of long-term immersion in the novel gargle on aligner mechanics was examined under simulated extreme conditions. The results showed that after immersion for up to 24 h at 37 °C, no significant differences were observed in any mechanical parameters compared with the control group. The tested parameters included elastic modulus, yield stress, yield strain, tensile strength, fracture strain, and stress-relaxation rate. These findings indicate that even under extended exposure, the novel gargle did not compromise the mechanical integrity or functional performance of clear aligners.

### 2.6 Evaluation of biocompatibility

Biocompatibility assessment constitutes a fundamental prerequisite in the development of any oral bioactive material, and novel gargle formulations are no exception. *Lonicera japonica*, a traditional herbal ingredient, has been demonstrated to be safe for long-term oral application. In this study, the biocompatibility of DMAHDM was comprehensively evaluated through both *in vitro* and *in vivo* investigations.

**Fig. 7:**
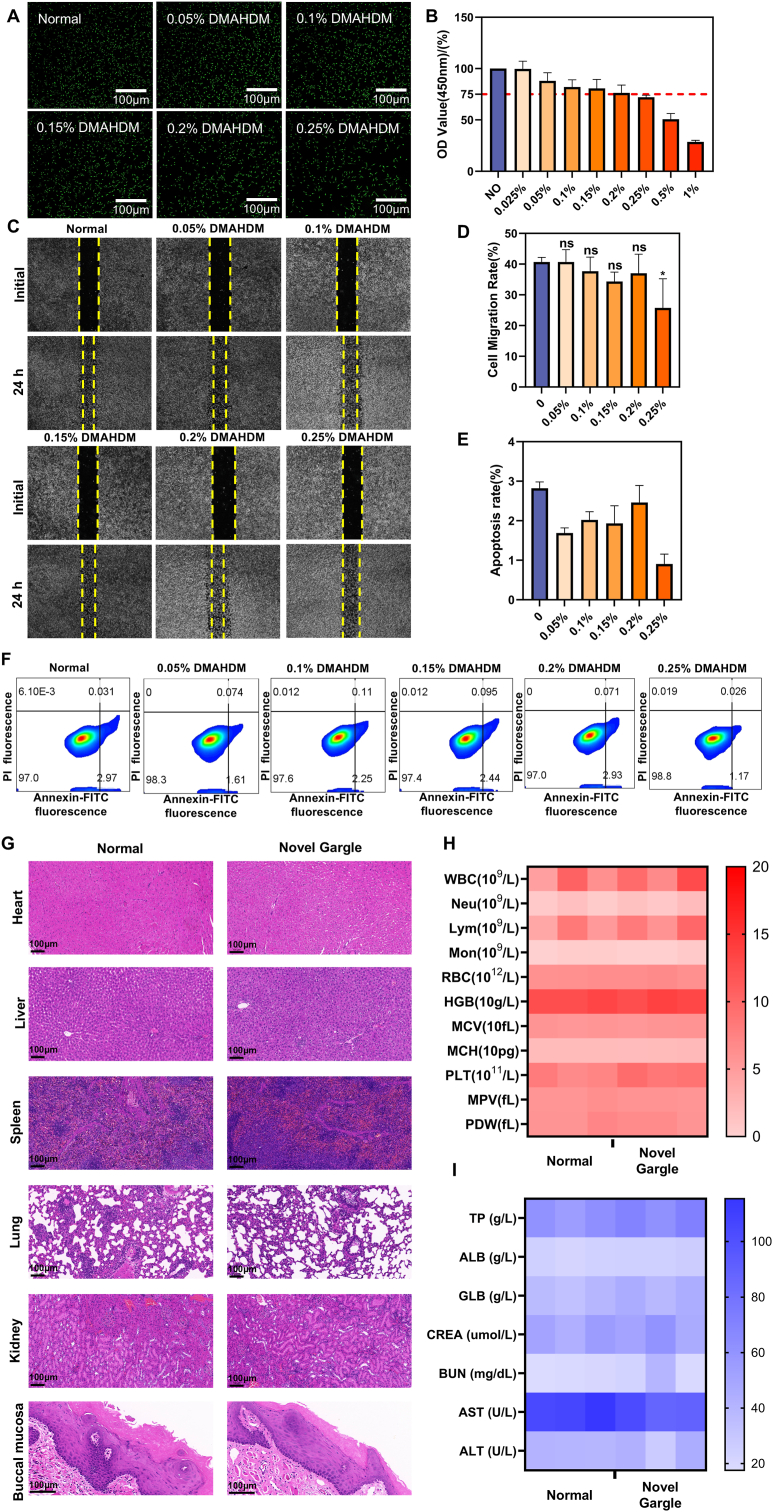
Evaluation of biocompatibility of the novel gargle. **A.** Fluorescence staining images of L929 fibroblasts. **B.** Cell proliferation measured by the Cell Counting Kit-8 (CCK-8) assay; the red dashed line indicates 75% cell viability. **C–D.** Representative scratch wound images and quantitative analysis of the wound-closure rate. **E–F.** Quantitative assessment of apoptosis rate and representative flow cytometry plots. **G.** Hematoxylin and eosin (H&E)-stained histological sections of rat organs and buccal mucosa after 60 days of oral exposure to the gargle. **H.** Hematological parameters. **I.** Serum biochemical indices of liver and kidney function. Data are presented as mean ± standard deviation (s.d.). For *in vitro* experiments, n ≥ 3; for *in vivo* experiments, n = 6. Statistical significance was analyzed using one-way analysis of variance (ANOVA) followed by Tukey's post hoc test. *p* < 0.05 (*), *p* < 0.01 (**), *p* < 0.001 (***), *p* < 0.0001 (****). (For interpretation of the references to colour in this figure legend, the reader is referred to the Web version of this article.)

At the cellular level, the cytotoxicity of DMAHDM at different mass fractions (0.05–0.25 wt%) was assessed using the CCK-8 assay. Fluorescence staining images (Fig. 7A) initially revealed that when the DMAHDM mass fraction was below 0.2 wt%, no obvious reduction in viable cell number was observed. Quantitative CCK-8 analysis (Fig. 7B) further confirmed that cell proliferation was significantly inhibited when the DMAHDM mass fraction reached 0.25 wt%, with viability dropping below 75% of the normal control. Therefore, DMAHDM at mass fractions of 0.25 wt% and below was selected for subsequent experiments.

A scratch wound-healing assay was further performed to evaluate the influence of DMAHDM on cell migration. As illustrated in Fig. 7C, the wound closure rate was not significantly affected when the DMAHDM mass fraction was ≤0.2 wt%. However, at 0.25 wt%, the healing rate was clearly reduced (Fig. 7D), consistent with the CCK-8 findings. Moreover, apoptosis analysis (Fig. 7E and F) demonstrated that DMAHDM at the tested mass fractions did not induce significant apoptotic activity. Collectively, these results indicate that 0.2 wt% represents the upper safety threshold for DMAHDM, beyond which potential cytotoxic effects may occur.

Based on these *in vitro* results, a gargle formulation containing 0.2 wt% DMAHDM was prepared for *in vivo* evaluation. The formulation was administered to rats through simulated oral rinsing for 60 consecutive days to mimic clinical usage. Throughout the experimental period, no abnormal behaviors, weight loss, or clinical symptoms were observed, suggesting excellent systemic tolerance. After euthanasia, major organs including the heart, liver, spleen, lungs, and kidneys were collected for histopathological and biochemical analyses. Hematoxylin–eosin (HE) staining (Fig. 7G) revealed intact tissue architecture with no inflammatory infiltration, necrosis, or other pathological alterations, indicating the absence of systemic toxicity.

Hematological (Fig. 7H) and biochemical (Fig. 7I) parameters further corroborated the histological findings. No statistically significant differences were observed between the saline control and gargle-treated groups. The hematological indices included white blood cells (WBC), neutrophils (Neu), lymphocytes (Lym), monocytes (Mon), red blood cells (RBC), hemoglobin (HGB), mean corpuscular volume (MCV), mean corpuscular hemoglobin (MCH), platelets (PLT), mean platelet volume (MPV), and platelet distribution width (PDW). In addition, biochemical markers of hepatic and renal function, including total protein (TP), albumin (Alb), globulin (Glb), creatinine (CREA), blood urea nitrogen (BUN), aspartate aminotransferase (AST), and alanine aminotransferase (ALT), remained within normal physiological ranges.

Taken together, these *in vitro* and *in vivo* findings demonstrate that the DMAHDM-containing gargle exhibits excellent cytocompatibility and biocompatibility, highlighting its potential for further preclinical and clinical translation. Compared with conventional mouthwashes such as chlorhexidine and CPC, which exert broad-spectrum, non-selective antibacterial effects associated with oral microbiota disruption and aesthetic concerns including staining of tooth surfaces and clear aligners, the present formulation appears to offer a more ecologically favorable profile. The incorporation of DMAHDM selectively reduces acidogenic bacteria while preserving commensal microbiota, and LJE provides complementary anti-inflammatory effects through modulation of the PPAR signaling pathway, representing mechanistic advantages over existing commercial agents. It should be acknowledged, however, that no commercial mouthwash was included as a positive control in the present study, and the comparison above is therefore based on mechanistic differences and published literature rather than direct experimental evidence.

## 3 Conclusion

The multifunctional gargle formulated with 0.1 wt% LJE and 0.2 wt% DMAHDM exhibits potent antibacterial efficacy, microbiome-regulating capability, anti-inflammatory activity, and protective effects against enamel demineralization, while demonstrating excellent biocompatibility and no adverse impact on the mechanical properties of clear aligners.

Collectively, these findings indicate that the novel multifunctional gargle offers a promising strategy for preventing multiple complications associated with orthodontic treatment and for maintaining overall oral health. Nevertheless, further clinical investigations are warranted to validate its therapeutic efficacy and to substantiate the translational potential of this work.

## 4 Experimental section

This study was approved by the Animal Ethics and Welfare Committee of Beijing Stomatological Hospital, Capital Medical University (Approval No. KQYY-202403-006).

### 4.1 Synthesis of DMAHDM and preparation of *Lonicera japonica* extract

DMAHDM was synthesized via a modified Menschutkin reaction following a previously reported procedure [40]. The successful synthesis was verified using Fourier-transform infrared (FTIR) spectroscopy and proton nuclear magnetic resonance (^1^H NMR) spectroscopy.

The *Lonicera japonica* extract was obtained from Xi'an Dalven Biotechnology Co., Ltd. (Xi'an, China). The extract was characterized by FTIR and ^1^H NMR spectroscopy to identify its principal active functional groups.

### 4.2 Evaluation of antibacterial properties

#### 4.2.1 Establishment of an in vitro multispecies plaque biofilm model

An *in vitro* multispecies plaque biofilm model was established according to previously reported methods [41]. Briefly, mixed human saliva samples were inoculated into McBain medium containing a defined proportion of sucrose to facilitate biofilm formation. The pH of 5 mL of supernatant collected from each group was measured at 8, 24, 48, and 72 h using a calibrated pH meter to monitor acidogenic activity within the biofilm.

#### 4.2.2 Live/dead bacterial viability staining

After 48 h of cultivation, the biofilm formed at the bottom of the culture dish. The culture medium was discarded, and the biofilm was gently rinsed with PBS. It was then immersed in either physiological saline or the novel gargle for 3 min. Bacterial viability was assessed using a LIVE/DEAD BacLight Bacterial Viability Kit (Invitrogen, Carlsbad, CA, USA) according to the manufacturer's protocol. Fluorescence microscopy was employed to visualize live (green fluorescence) and dead (red fluorescence) bacterial populations within the biofilm.

#### 4.2.3 Evaluation of biofilm metabolic activity

The metabolic activity of the biofilm was quantified using an MTT Cell Proliferation and Cytotoxicity Assay Kit (Beyotime, Shanghai, China) according to the manufacturer's protocol. The optical density (OD) at 450 nm was measured using a microplate reader, and the metabolic activity was normalized to the surface area of the culture well (A_450_/cm^2^).

#### 4.2.4 Colony-forming unit (CFU) counting

Biofilms were harvested by ultrasonic agitation followed by vortex mixing to ensure complete dispersion. Serial dilutions of the biofilm suspensions were plated on tryptic soy agar (TSA) supplemented with 5% defibrinated sheep blood for total bacterial counts. After incubation under aerobic conditions at 37 °C for 48 h, the number of colony-forming units (CFUs) was determined and expressed as log_10_ CFU/mL.

#### 4.2.5 High-throughput 16S ribosomal RNA (16S rRNA) gene sequencing

Fifteen male SD rats (6–8 weeks old, 250–350 g) were randomly divided into three groups (n = 5 per group): control, periodontitis, and gargle. After one week of acclimatization, periodontitis was induced in the periodontitis and gargle groups using the ligature method, and simulated mouth-rinsing was performed for 21 consecutive days as described above.

At the end of the experimental period, oral bacterial samples were collected using sterile sampling swabs. High-throughput 16S ribosomal RNA (rRNA) gene sequencing was performed by OE Biotech Co., Ltd. (Shanghai, China). Briefly, genomic DNA was extracted using a MagPure Soil DNA LQ Kit (Magan, Guangzhou, China) according to the manufacturer's protocol. The hypervariable V3–V4 regions of the bacterial 16S rRNA gene were amplified with universal primers, and sequencing was carried out on an Illumina NovaSeq 6000 platform (Illumina, San Diego, CA, USA).

The resulting sequencing data were processed to analyze the microbial community composition, α-diversity (Chao1, Shannon, and Simpson indices), and β-diversity by principal coordinate analysis (PCoA). Taxonomic classification was performed at the phylum level to compare microbial profiles among the different groups.

### 4.3 Evaluation of anti-inflammatory properties

#### 4.3.1 *In vitro* evaluation of anti-inflammatory properties

Based on the biocompatibility assessment, DMAHDM was used at a mass fraction of 0.2 wt%, while LJE was prepared at mass fractions of 0.03 wt%, 0.06 wt%, 0.09 wt%, and 0.12 wt% in the culture medium. Rat macrophage RAW264.7 cells (passages 3–6) were seeded into six-well plates at a density of 1.0 × 10^6^ cells per well and cultured for 24 h at 37 °C in a humidified atmosphere containing 5% CO_2_. The medium was then replaced with fresh medium containing the respective mass fractions of LJE and DMAHDM. To induce inflammation, lipopolysaccharide (LPS; Sigma–Aldrich, St. Louis, MO, USA) was added to both the positive control and experimental groups at a final concentration of 1 μg/mL for 24 h.

##### 4.3.1.1 Reverse transcription quantitative polymerase chain reaction (RT-qPCR)

Total RNA was extracted using an RNA extraction kit (Vazyme, Nanjing, China) according to the manufacturer's protocol. The isolated RNA was reverse-transcribed into complementary DNA (cDNA), and quantitative real-time polymerase chain reaction (RT–qPCR) was performed using reagents from Takara Bio Inc. (Shiga, Japan) to evaluate the relative expression levels of tumor necrosis factor-α (*Tnfa*) and interleukin-6 (*Il6*). The primer sequences used for amplification are listed in Table S1.

##### 4.3.1.2 Enzyme-linked immunosorbent assay (ELISA)

After 24 h of LPS stimulation, the culture supernatants were collected, and the protein levels of TNF-α and IL-6 were quantified using enzyme-linked immunosorbent assay (ELISA) kits (Cloud-Clone, Wuhan, China) according to the manufacturer's protocol.

#### 4.3.2 In vivo evaluation of anti-inflammatory properties

Following the *in vitro* experiments, a gargle formulation containing 0.2 wt% DMAHDM and 0.1 wt% LJE was prepared in sterile water for subsequent *in vivo* evaluation.

##### 4.3.2.1 Animal model establishment

Twelve male SD rats (6–8 weeks old, 250–350 g) were randomly divided into three groups (n = 4 per group): control, periodontitis, and gargle. After one week of acclimatization, periodontitis was induced in the periodontitis and gargle groups using the ligature method [42]. Under anesthesia, a 4-0 nonabsorbable silk ligature was carefully placed around the bilateral first and second molars and between the second and third molars, and then securely tied on the palatal side. The rats were maintained under standard laboratory conditions for 21 days, with daily monitoring of ligature displacement; any dislodged ligatures were replaced immediately. After 21 days, the ligatures were removed, and the animals were subjected to subsequent analyses.

##### 4.3.2.2 Micro-computed tomography (Micro-CT)

After successful establishment of the periodontitis model, the control and periodontitis groups were treated with saline, whereas the gargle group received the novel gargle formulation. Following 21 days of simulated rinsing, the maxillary alveolar bone was scanned using a desktop micro-computed tomography (micro-CT) system. Three-dimensional images were reconstructed to evaluate alveolar bone resorption. The extent of bone loss was quantified as the mean distance between the cementoenamel junction (CEJ) and the alveolar bone crest (ABC) at the mesial, central, and distal sites of the maxillary second molar.

##### 4.3.2.3 Hematoxylin and eosin (H&E) staining

Following euthanasia, the maxillary bone specimens were fixed in 4% paraformaldehyde and decalcified in 10% ethylenediaminetetraacetic acid (EDTA) solution at 4 °C for four weeks. The samples were then dehydrated, embedded in paraffin, sectioned at a thickness of 5 μm, and stained with hematoxylin and eosin (H&E) for histopathological evaluation.

##### 4.3.2.4 RNA sequencing (RNA-seq)

All rats were euthanized, and gingival tissues from both sides were harvested and immediately snap-frozen in liquid nitrogen to prevent RNA degradation. Portions of the samples were subsequently used for RT–qPCR analysis.

RNA sequencing (RNA-seq) library construction and sequencing were performed by OE Biotech Co., Ltd. (Shanghai, China). Gene expression levels were quantified as fragments per kilobase of transcript per million mapped reads (FPKM) using HTSeq-count. Differentially expressed genes (DEGs) were identified using the DESeq2 package, with significance thresholds set at *p* < 0.05 and |log_2_ fold change| > 1. Gene Ontology (GO) enrichment analysis was conducted to annotate the functional roles of DEGs, and the Kyoto Encyclopedia of Genes and Genomes (KEGG) database was applied to identify significantly enriched signaling pathways.

### 4.4 *In vivo* evaluation of the protective effect against enamel demineralization

#### 4.4.1 Establishment of the animal model

Human first premolars extracted for orthodontic purposes were obtained from the Beijing Stomatology Hospital, Capital Medical University (Beijing, China). Soft debris on the crown surfaces was removed using a sterile scalpel, and the teeth were immersed in 3% sodium hypochlorite solution to eliminate adherent microorganisms. The enamel was sectioned using a diamond saw to obtain slices approximately 3 × 3 × 1 mm in size. Each slice was embedded in acrylic resin to form a circular disc (approximately 4 mm in diameter and 1 mm in thickness). Two small holes were drilled in the resin base to allow fixation with ligature wire during intraoral placement in rats [43].

Ten male SD rats (6–8 weeks old, 250–350 g) were randomly divided into two groups (n = 5 per group). After one week of acclimatization, the enamel specimens were fixed onto the buccal mucosa of each rat. Subsequently, 0.2 mL of *Streptococcus mutans* suspension (1 × 10^8^ CFU/mL) was applied to the enamel slices, tongue, and buccal mucosa to establish an *in vivo* demineralization model. Rats were fed a cariogenic diet 2000 (Trophic Animal Feed High-Tech Co., Ltd., Nantong, China) and provided with 5% sucrose water *ad libitum*. To maintain bacterial colonization, 0.2 mL of *S. mutans* suspension was reapplied weekly. After four weeks, all animals were euthanized, and the human enamel specimens were carefully retrieved for further analysis.

#### 4.4.2 Scanning electron microscopy (SEM) and energy-dispersive X-ray spectroscopy (EDS)

After cleaning, the enamel specimens were sputter-coated with a thin layer of gold (10 mA) and examined under a scanning electron microscope (SEM) to observe surface morphology. The elemental composition of the enamel was analyzed using energy-dispersive X-ray spectroscopy (EDS) operated at an accelerating voltage of 15 kV and a beam current of 6.4 nA. The weight percentages of calcium (Ca) and phosphorus (P) were determined semi-quantitatively to evaluate the degree of mineral loss.

#### 4.4.3 Nanoindentation test

To evaluate the mechanical properties of the enamel, each specimen was embedded in a resin block with the enamel surface exposed. Mechanical characterization was conducted using a nanoindenter (Hysitron TriboIndenter, Bruker, Minneapolis, MN, USA) equipped with a Berkovich diamond tip. The maximum load was set to 25 mN, with a loading time of 15 s and a holding time of 10 s at the peak load. The elastic modulus (E) and hardness (H) of each specimen were automatically calculated from the load–displacement curves.

### 4.5 Evaluation of the effect on orthodontic appliance mechanical properties

#### 4.5.1 Preparation of specimens

The commonly used thermoplastic materials for clear orthodontic aligners are thermoplastic polyurethane (TPU) and polyethylene terephthalate glycol-modified (PETG). In accordance with the national standard GB/T 1040.1–2018 and the industry standard YY/T 1819–2022, twelve dumbbell-shaped specimens of each material were fabricated by precision cutting.

#### 4.5.2 Uniaxial tensile test and stress relaxation test

After immersion, the specimens were mounted on a universal testing machine (Instron 3369, Instron Corp., Norwood, MA, USA) using threaded grips, and an extensometer was attached to calibrate strain measurements. A uniaxial tensile test was then performed at a crosshead speed of 0.01 mm/s. The stress–strain and load–displacement curves were recorded in real time to determine the elastic modulus, yield stress, yield strain, ultimate tensile strength, and fracture elongation. The test automatically terminated upon specimen fracture, and complete stress–strain data were exported for subsequent analysis.

For the stress relaxation test, additional specimens were evaluated under the same grouping conditions. After reaching the predetermined initial strain (ε_0_), the system automatically switched to strain-hold mode (t = 0), maintaining the extensometer gauge length constant throughout the 3 h relaxation period. The instrument continuously recorded the stress decay curve, from which the stress relaxation rate was calculated.

### 4.6 Evaluation of biocompatibility

The primary active components of the gargle were DMAHDM and LJE powder. LJE, a food-grade additive, is generally recognized as nontoxic. Based on previous findings that DMAHDM exhibits potent antibacterial activity at a mass fraction of 0.15 wt%, five mass fractions (0.05 wt%, 0.10 wt%, 0.15 wt%, 0.20 wt%, and 0.25 wt%) were selected for *in vitro* biocompatibility testing to determine the maximum safe level for subsequent *in vivo* experiments. The mass fraction of LJE was fixed at 0.1 wt%.

#### 4.6.1 Cell proliferation assay

Mouse fibroblast L929 cells were used to evaluate the effects of different gargle samples on cell proliferation. Third-passage L929 fibroblasts were cultured in media containing various mass fractions of DMAHDM. Cells were seeded into 96-well plates at a density of 6.0 × 10^3^ cells per well and incubated for 24 h. Cell proliferation was assessed using a Cell Counting Kit-8 (CCK-8; Selleck, Houston, TX, USA) according to the manufacturer's instructions. In addition, after 24 h of incubation, the cells were fixed with 4% paraformaldehyde and stained with SYTO 9 (Beyotime, Shanghai, China) for fluorescence imaging to observe cell morphology and growth.

#### 4.6.2 Cell migration assay

L929 cells were seeded into six-well plates at a density of 1.0 × 10^6^ cells per well and incubated for 24 h under serum-free conditions. A sterile 1-mL pipette tip was then used to create a vertical scratch across the cell monolayer. Detached cells were gently removed with phosphate-buffered saline (PBS; Biosharp, Anhui, China), and fresh culture medium containing different mass fractions of DMAHDM was added. Three parallel wells were prepared for each group. Images of the wound area were captured at 0 and 24 h to evaluate the effect of DMAHDM mass fraction on cell migration.

#### 4.6.3 Apoptosis assay

L929 cells were cultured with the respective extraction solutions for 48 h. Apoptosis was assessed using an Annexin V–FITC apoptosis detection kit (Beyotime, Shanghai, China) according to the manufacturer's protocol, followed by flow cytometric analysis.

#### 4.6.4 In vivo evaluation of biocompatibility

Twelve male Sprague–Dawley (SD) rats (6–8 weeks old, 250–350 g) were acclimated for one week and then randomly divided into two groups (n = 6 per group): saline control and gargle treatment. All animals were maintained under standard laboratory conditions with *ad libitum* access to food and water. Simulated mouth-rinsing was conducted at 08:00, 14:00, and 20:00 each day by gently flushing the oral cavity with 5 mL of either saline or the novel gargle (containing 0.2 wt% DMAHDM and 0.1 wt% LJE) using a sterile disposable syringe. The solution was retained in the oral cavity for approximately 60 s before natural expectoration to simulate the gargling action.

After 60 consecutive days of treatment, the rats were anesthetized, and blood samples were collected from the abdominal aorta. Approximately 3 mL of arterial blood was obtained in both EDTA-coated tubes and gel-separation tubes. Samples in EDTA tubes were stored at 4 °C for hematological analysis, whereas serum was isolated by centrifugation at 4000 rpm for 10 min after standing at room temperature for 1 h and subsequently stored at −20 °C. Following blood collection, the rats were euthanized, and the buccal mucosa and major organs (heart, liver, spleen, lungs, and kidneys) were excised and fixed in 4% paraformaldehyde for 24 h.

Routine hematological parameters were analyzed using an automated hematology analyzer, and serum biochemical indices of liver and kidney function were measured with a biochemical analyzer. Paraffin-embedded tissues were sectioned and stained with hematoxylin and eosin (H&E) for histopathological examination. Digital images were captured using a slide scanner and analyzed with *SlideViewer* 2.6 software (3DHISTECH, Budapest, Hungary).

### 4.7 Statistical analysis

All statistical analyses were performed using Statistical Package for the Social Sciences (SPSS, version 26; IBM Corp., Armonk, NY, USA). Quantitative data are expressed as mean ± standard deviation (SD). Prior to statistical analysis, normality of data distribution was assessed using the Shapiro-Wilk test; all datasets were found to conform to a normal distribution (*p* > 0.05). Differences between two groups were analyzed using an independent-samples *t*-test, while comparisons among multiple groups were conducted using one-way analysis of variance (ANOVA) followed by Tukey's post hoc test. A p-value <0.05 was considered statistically significant. Statistical significance in figures is denoted as follows: *p < 0.05* (*)*, p < 0.01* (**)*, p < 0.001* (***), and *p* < 0.0001 (****).

## CRediT authorship contribution statement

**Yuqing Zhang:** Formal analysis, Investigation, Writing – original draft. **Bo Wang:** Formal analysis, Investigation, Writing – original draft. **Ce Bian:** Formal analysis, Investigation, Writing – original draft. **Chaoran Yu:** Data curation, Validation, Visualization. **Mengyao Zhu:** Data curation, Validation, Visualization. **Yiman Guo:** Data curation, Validation, Visualization. **Honglei Yue:** Supervision, Writing – review & editing. **Wenting Yu:** Supervision, Writing – review & editing. **Yuxing Bai:** Conceptualization, Methodology, Project administration, Supervision, Writing – review & editing. **Ning Zhang:** Conceptualization, Methodology, Project administration, Supervision, Writing – review & editing.

## Declaration of competing interest

The authors declare no conflicts of interest.

## Data Availability

Data will be made available on request.
