# Supplementary material for: A DMAHDM–herbal hybrid gargle for orthodontic-associated complications via oral microbiota regulation, inflammation inhibition, and enamel protection
Source: Mater Today Bio. 2026 Jun 22;39:103372. doi: 10.1016/j.mtbio.2026.103372 (PMC13320463; doi:10.1016/j.mtbio.2026.103372)
Supplement: Multimedia component 1 [file mmc1.docx]

**A DMAHDM–Herbal Hybrid Gargle for Orthodontic-Associated Complications via Oral Microbiota Regulation, Inflammation Inhibition, and Enamel Protection**

Yuqing Zhang¹^,^**^#^**, Bo Wang²^,^**^#^**, Ce Bian¹^,^**^#^**, Chaoran Yu¹, Mengyao Zhu¹, Yiman Guo¹, Honglei Yue³, Wenting Yu¹^,^**^*^**, Yuxing Bai¹^,^**^*^**, Ning Zhang¹^,^**^*^**

**Affiliations**

¹ Department of Orthodontics, Beijing Stomatological Hospital and School of Stomatology, Capital Medical University, No.9 Fanjiacun Road, Fengtai District, Beijing, 100070, PR China

² Department of Stomatology, The First Affiliated Hospital of Dalian Medical University, No. 222 Zhongshan Road, Dalian 116000, Liaoning, PR China

³ Beijing Institute of Dental Research, Beijing Stomatological Hospital, Capital Medical University, No. 9 Fanjiacun Road, Fengtai District, Beijing, 100070, PR China

**Supplementary Table**

**Supplementary table 1.** Primer sequences of target and reference genes used for RT–qPCR analysis.

| Gene | Primer sequence (5′–3′) |
| --- | --- |
| Rat |  |
| *Il6* | F: GCCCACCAGGAACGAAAGTC |
|  | R: TGGCTGGAAGTCTCTTGCGG |
| *Tnfa* | F: GATCGGTCCCAACAAGGAGG |
|  | R: CTTGGTGGTTTGCTACGACG |
| *Gapdh* | F: CCGCATCTTCTTGTGCAGTG |
|  | R: CGATACGGCCAAATCCGTTC |
| Mouse |  |
| *Il6* | F: TGCTGGTGACAACCACGGCC |
|  | R: GTACTCCAGAAGACCAGAGG |
| *Tnfa* | F: CCTGTAGCCCACGTCGTAGC |
|  | R: TTGACCTCAGCGCTGAGTTG |
| *Gapdh* | F: GGTTGTCTCCTGCGACTTCA |
|  | R: GGTGGTCCAGGGTTTCTTACTC |

**Supplementary Figures**

**
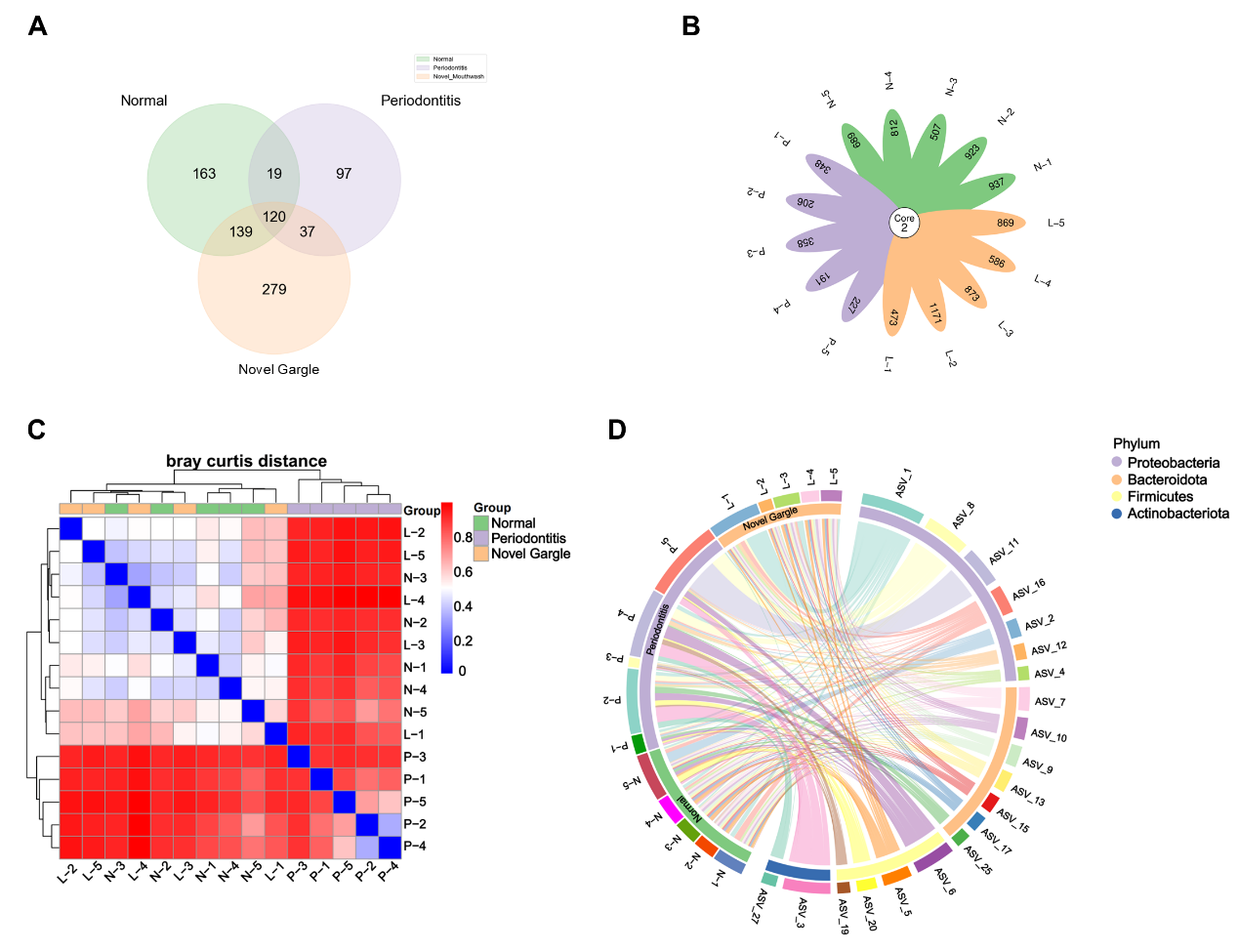
**

**Supplementary Figure 1** Supplementary data for oral microbiome analysis. **A.** Venn diagram of shared and unique microbial species across groups. **B.** Petal plot showing the distribution of species across groups. **C.** Beta-diversity analysis based on Bray–Curtis distance matrix. **D.** Relative abundance of dominant bacterial phyla at the phylum level (supplementary).


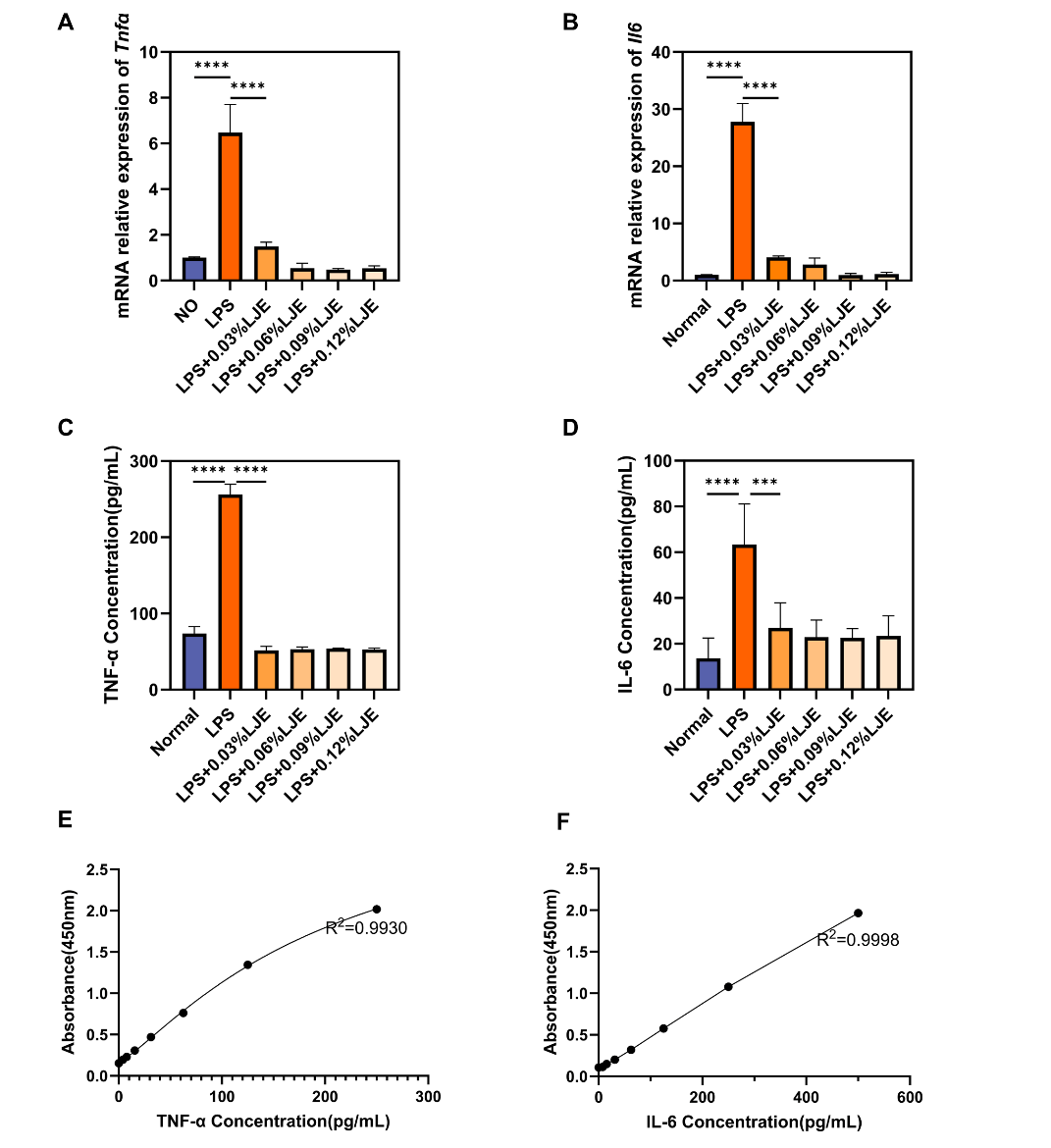


**Supplementary Figure 2** Evaluation of the anti-inflammatory effects of the novel gargle *in vitro*. **A–B.** Relative mRNA expression of *Tnfa* and *Il6* in RAW264.7 macrophages. **C–D.** Protein concentrations of TNF-α and IL-6 measured by enzyme-linked immunosorbent assay (ELISA). **E–F.** ELISA standard curves for TNF-α and IL-6, respectively. For all experiments, n ≥ 3. Data are presented as mean ± standard deviation (s.d.). Statistical significance was analyzed using one-way analysis of variance (ANOVA) followed by Tukey's post hoc test. *p* < 0.05 (*), *p* < 0.01 (**), *p* < 0.001 (***), *p* < 0.0001 (****).


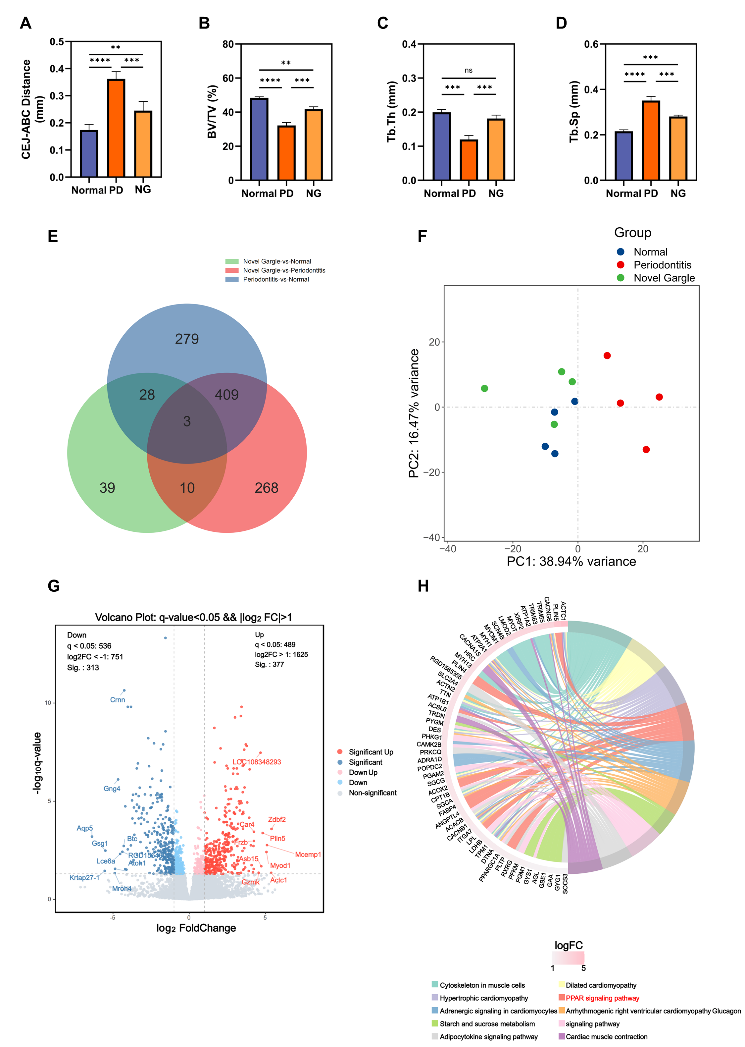


**Supplementary Figure 3** Evaluation of the anti-inflammatory effects and mechanism of the novel gargle *in vivo*. **A.** Quantitative assessment of the cementoenamel junction–alveolar bone crest (CEJ-ABC) distance. **B–D.** Quantitative micro-CT analysis of trabecular bone microstructural parameters, including bone volume fraction (BV/TV), trabecular thickness (Tb.Th), and trabecular separation (Tb.Sp). **E.** Venn diagram of differentially expressed genes (DEGs) across comparisons. **F.** Principal component analysis (PCA) of RNA sequencing (RNA-seq) data. **G.** Volcano plot of DEGs between the periodontitis and novel gargle groups. **H.** Chord diagram illustrating the associations between differentially enriched Kyoto Encyclopedia of Genes and Genomes (KEGG) pathways and functional categories. For all experiments, n = 4. Data are presented as mean ± standard deviation (s.d.). Statistical significance was analyzed using one-way analysis of variance (ANOVA) followed by Tukey's post hoc test. *p* < 0.05 (*), *p* < 0.01 (**), *p* < 0.001 (***), *p* < 0.0001 (****).


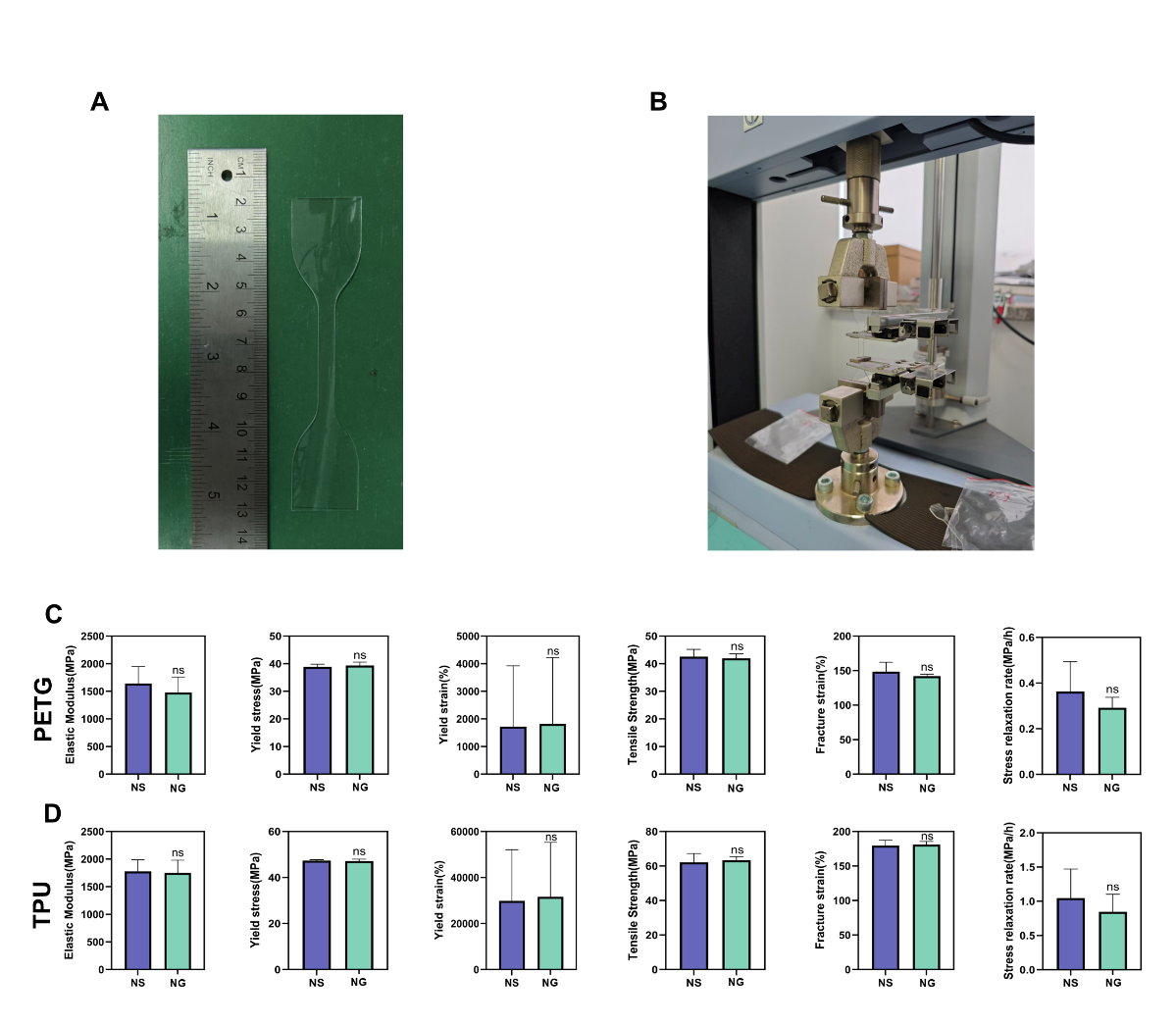


**Supplementary Figure 4** Evaluation of material compatibility of the novel gargle. **A.** Specimen used for mechanical testing. **B.** Universal testing machine used for mechanical measurements. **C-D.** Mechanical parameters, including elastic modulus, yield stress, yield strain, tensile strength, fracture strain, and stress-relaxation rate, remained stable for polyethylene terephthalate glycol-modified (PETG) and thermoplastic polyurethane (TPU) clear aligner materials. n ≥ 6. Data are presented as mean ± standard deviation (s.d.). Statistical significance was analyzed using one-way analysis of variance (ANOVA) followed by Tukey’s post-hoc test. *p* < 0.05 (*)*, p < 0.01* (**)*, p < 0.001* (***), *p* < 0.0001 (****).
